# A Single-Step Genome Wide Association Study on Body Size Traits Using Imputation-Based Whole-Genome Sequence Data in Yorkshire Pigs

**DOI:** 10.3389/fgene.2021.629049

**Published:** 2021-07-02

**Authors:** Huatao Liu, Hailiang Song, Yifan Jiang, Yao Jiang, Fengxia Zhang, Yibing Liu, Yong Shi, Xiangdong Ding, Chuduan Wang

**Affiliations:** National Engineering Laboratory for Animal Breeding, Laboratory of Animal Genetics, Breeding and Reproduction, Ministry of Agriculture, College of Animal Science and Technology, China Agricultural University, Beijing, China

**Keywords:** pigs, body size traits, ssGWAS, two-trait model, SNP effect

## Abstract

The body shape of a pig is the most direct production index, which can fully reflect the pig’s growth status and is closely related to important economic traits. In this study, a genome-wide association study on seven body size traits, the body length (BL), height (BH), chest circumference (CC), abdominal circumference (AC), cannon bone circumference (CBC), rump width (RW), and chest width (CW), were conducted in Yorkshire pigs. Illumina Porcine 80K SNP chips were used to genotype 589 of 5,572 Yorkshire pigs with body size records, and then the chip data was imputed to sequencing data. After quality control of imputed sequencing data, 784,267 SNPs were obtained, and the averaged linkage disequilibrium (*r*^2^) was 0.191. We used the single-trait model and the two-trait model to conduct single-step genome wide association study (ssGWAS) on seven body size traits; a total of 198 significant SNPS were finally identified according to the *P*-value and the contribution to the genetic variance of individual SNP. 11 candidate genes (*CDH13*, *SIL1*, *CDC14A*, *TMRPSS15*, *TRAPPC9*, *CTNND2*, *KDM6B*, *CHD3*, *MUC13*, *MAPK4*, and *HMGA1*) were found to be associated with body size traits in pigs; *KDM6B* and *CHD3* jointly affect AC and CC, and *MUC13* jointly affect RW and CW. These genes are involved in the regulation of bone growth and development as well as the absorption of nutrients and are associated with obesity. *HMGA1* is proposed as a strong candidate gene for body size traits because of its important function and high consistency with other studies regarding the regulation of body size traits. Our results could provide valuable information for pig breeding based on molecular breeding.

## Introduction

Pork is widely used as an important animal protein resource and has become one of the main sources of human protein. Commercial pigs (e.g., Duroc, Yorkshire, and Landrace pigs) have the characteristics of fast growth, high feed utilization rate, high lean meat rate, and obvious economic benefits. Therefore, it is not only used for a large number of breeding production, but also has been the focus of research. The body size trait is an important phenotypic trait that can reflect the overall appearance of animals. Compared with the description of physical appearance, body size traits can objectively reflect the response of pigs to their environment and other aspects (Ohnishi and Satoh, 2018). In pig breeding, the body shape character index is often used as the most direct production index of a pig. Body size is a typical quantitative (or complex) trait; understanding the genetic mechanism of body size differences among individuals can effectively help control the growth and production of animals (Niu et al., 2013). At present, there is a large amount of research on genetic parameters of pig external traits, which accelerate the process of genetic improvement of related traits. With the development of molecular biotechnology, many studies have been carried out to clarify the genetic basis of pig body size traits.

So far, 1172 QTLs have been found related to body size traits in pigs according to PigQTLdb database^1^. Although a range of research has been done in QTL mapping, wide confidence intervals (covering more than 20 CM) for the positions of QTL remain that have rarely been replicated (Schreiweis et al., 2005; Soller et al., 2006). A new research era was initiated with advances in single nucleotide polymorphism (SNP) chip and sequencing technology, and genome wide association study (GWAS) has become one of the most efficient methods to detect genetic variation in livestock (Mackay et al., 2009). Compared with traditional QTL localization, GWAS has more advantages in mining the intensity of medium-potency variation sites and defining the accuracy of genome segments containing variation sites (Risch, 1996; Hirschhorn and Daly, 2005; Klein et al., 2005; Simon-Sanchez et al., 2009). Although a large number of genome-wide association studies have been carried out in pigs, only a few GWAS focused on identifying genes related to external traits. In particular, the investigation on body height, cannon bone circumference, rump width, and other important body size traits are still lacking.

Marker density is one key factor affecting the efficiency of GWAS as gene mapping mainly relies on the linkage disequilibrium between causal mutation and markers (Brondum et al., 2012). Whole genome sequence data can definitely meet such requirements. In recent years, with the rapid development of the new generation of sequencing technology, the cost of sequencing has been reduced rapidly. On one hand, the large number of samples and the subsequent processing of sequence data are still time-consuming and costly, limiting its utilization in genetic analysis. On the other hand, genotype imputation provides an efficient tool to improve the marker density of SNP chips based on sequence data. It can accurately predict the genotypes of polymorphic sites not covered by the widely used SNP chip, allowing more genetic loci to be applied to association analysis and improving the possibility of discovering new pathogenic genes (Marchini and Howie, 2010; van Leeuwen et al., 2015). In this study, we used imputation-based whole genome sequence data to carry out GWAS on seven body size traits in pigs.

## Materials and Methods

### Ethics Statement

The whole recording procedure of ear tissue samples was carried out in strict accordance with the protocol approved by the Institutional Animal Care and Use Committee (IACUC) at the China Agricultural University. The IACUC of the China Agricultural University approved this study (permit number DK996).

### Animals and Phenotypes

Yorkshire pigs born from 2013-2016 from one pig breeding farm in Beijing were collected in this study. A performance test on seven body size traits were carried out at the body weight of about 100 kg for pigs. In total, 5,572 Yorkshire pigs with phenotypic records and pedigree information were selected. The seven body size traits included body length (BL), body height (BH), chest circumference (CC), abdominal circumference (AC), cannon bone circumference (CBC), chest width (CW), and rump width (RW). Table 1 presents the descriptive statistics of body weight and seven body size traits. There were 4898 records for AC and 5572 records for the other six body size traits and body weight. The Shapiro test function in the Stats package of R language was used to test if the phenotypic values of the seven body size traits followed normal distribution, and the results showed that all traits followed normal distribution. Body weight had the largest standard deviation of 12.59 kg and coefficient of variation of 12.43%; it was used as a covariate considering its influence on the body size traits in further analysis.

**TABLE 1:** Descriptive statistics for body weight and seven body size traits.

Trait^1^	N-obs^2^	Mean	S.D.	CV(%)	Min value	Max value
BL(cm)	5573	108.89	6.18	5.67	88	134
BH(cm)	5573	62.87	2.92	4.64	51	75
CC(cm)	5573	104.58	5.75	5.50	85	126
AC(cm)	4898	113.52	6.31	5.56	94	137
CW(cm)	5572	29.75	2.31	7.76	19	38
RW(cm)	5573	31.64	2.13	6.73	22	40
CBC(cm)	5573	17.98	1.03	5.73	13	23
BW(kg)	5573	101.31	12.59	12.43	61	150

*^1^BL, body length; BH, body height; CC, chest circumference; AC, abdominal circumference; CBC, cannon bone circumference; RW, rump width; CW, chest width. ^2^N-obs, number of observations.*

### Genotype Data and Imputation

In this study, 589 out of 5572 Yorkshire pigs with body size records were genotyped using the PorcineSNP80 Bead Chip (Illumina, San Diego, CA, United States), which includes 68,528 SNPs across the whole pig genome. In order to improve the marker density, the genotyped animals with another 6103 pigs genotyped with PorcineSNP80 (Song et al., 2019) were imputed to whole genome sequence data using Beagle 4.1 (Browning and Browning, 2009). A wide collection of 289 sequenced pigs all with average sequencing depths of ∼25X from six different pig breeds were used as reference data for imputation and each breed contained 24 to 94 pigs. The composition of reference data and the SNP calling of these individuals were described by Yan et al. (2017). After SNP calling, 46,766,110 SNPs were retained as the reference panel for imputation. On average, the genotype concordance rate across all variants was 92%, which is sufficient for further analysis (Song et al., 2019). After imputation, in this study, the following genotype quality control procedure was carried out using the PLINK software (v1.90) (Purcell et al., 2007). SNPs with minor allele frequency (MAF) lower than 0.01 and that deviated from the Hardy - Weinberg equilibrium (*P* < 10^–6^) were excluded and only variants located on autosomes were used for further analysis. SNP with call rates less than 0.95 were removed. Individuals with call rates less than 0.90 were excluded. In addition, in order to decrease the influence of the dependence of adjacent markers on the high false positive of GWAS analysis, the SNP were further pruned, and the SNP with linkage disequilibrium (*r*^2^) in slide window of 50 SNPs less than 0.9 were selected. Finally, all the genotyped animals and 784267 SNPs were retained.

### Statistical Models

#### Genetic Correlation

According to the information of 5,572 pigs in this study, the restricted maximum likelihood method (AI-REML) in DMU v6.0 software (Jensen and Madsen, 2000) was used to estimate the genetic correlations of seven body size traits.

The animal model was used to estimate the genetic parameters:

$$\text{y}=\mu +X\,\text{b}+{\text{Z}}_{1}\,\text{a}+{\text{Z}}_{2}\,\text{t}+\text{e},$$

with

$$\text{E}\,\left\{\begin{array}{c} \text{y}\\ \text{a}\\ \text{t}\\ e \end{array}\right\}={\left\{\begin{array}{c} \text{Xb}\\ 0\\ 0\\ 0 \end{array}\right\}}_{,}\,V\,a\,r\,\left\{\begin{array}{c} \text{a}\\ t\\ \text{e} \end{array}\right\}=\left\{ \begin{array}{c} \text{A}\,\sigma_{a}^{2} \end{array}\right.\,\begin{array}{c} 0\\ \text{I}\,\sigma_{t}^{2} \end{array}\,\begin{array}{c} 0\\ 0\\ \text{I}\,\sigma_{e}^{2} \end{array}\}$$

where, **y** is the vector of phenotypic values of each body size trait; **μ** is the population mean; **b** is the fixed effect of herd-year-season; **a** is the vector of additive genetic effects; **t** is the covariate vector of body weight effects; and **e** is a vector of residual error effects. **X**, **Z_**1**_,** and **Z_**2**_** are incidence matrices associating **b**, **a,** and **t** with **y**, respectively. **A** is the genetic relationship matrix, five generations of pedigree were traced back to construct A, and **σ_**a**_^**2**^** is the additive genetic variance. **I** is the identity matrix of appropriate dimension, **σ_**t**_^**2**^** is the variance of body weight effect, and **σ_**e**_^**2**^** is the residual variance.

As the following two-trait model was constructed, the same designs and similar methods were used to combine seven body size traits to construct a matrix equation to form a seven-trait model, which was mainly used to calculate the genetic correlation

$$\begin{array}{l} \left[\begin{array}{c} y_{1}\\ y_{2} \end{array}\right]\;=\left[\begin{array}{cc} X_{1} & 0\\ 0 & X_{2} \end{array}\right]\left[\begin{array}{c} b_{1}\\ b_{2} \end{array}\right]\;+\;\left[\begin{array}{cc} Z_{11} & 0\\ 0 & Z_{12} \end{array}\right]\left[\begin{array}{c} a_{1}\\ a_{2} \end{array}\right]\;\\ \;+\;\left[\begin{array}{cc} Z_{21} & 0\\ 0 & Z_{22} \end{array}\right]\left[\begin{array}{c} t_{1}\\ t_{2} \end{array}\right]+\left[\begin{array}{c} e_{1}\\ e_{2} \end{array}\right], \end{array}$$

where all letter representations are the same as the single trait model above and the subscript numbers 1 and 2 represent the two body size traits.

Subsequently, genetic correlations were calculated based on the variance components as follows:

$${\text{r}}_{A}=\frac{\text{cov}\,\left({\text{a}}_{1},{\text{a}}_{2}\right)}{\sigma_{a_{1}}\,\sigma_{a_{2}}}$$

where, r_*A*_ is the genetic correlation between trait 1 and trait 2, a_1_ and a_2_ represent the additive genetic values of trait 1 and trait 2 for the same individuals, cov (a1, a2) and σ_*a1*_, σ_*a2*_ refer to the genetic covariance of two traits and the genetic standard deviation of trait 1 and trait 2, respectively.

#### Genome-Wide Association Study

In this study, single-step GWAS (ssGWAS), which can simultaneously use all the SNP information and utilize the ungenotyped animals with phenotypic records (Wang et al., 2012), was implemented to identify significant SNPs associated with body size traits. Considering the genetic correlations between body size traits, the two-trait model is used for the traits with high genetic correlation, while the single-trait model is used for the rest of the traits.

##### Single-trait ssGWAS

The single-trait ssGWAS model was used for three body size traits: BL, BH, and CBC.

y=Xb+γ⁢W+Zg+e

where **y** is the vector of phenotypic values, **b** is the vector of fixed effects including herd-year-season-sex, **W** is the covariate of body weight, γ is the regression coefficient associating **W**, **g** is the vector of additive genetic effects, following a normal distribution of $N\,\left(0,\text{H}\,\sigma_{g}^{2}\right)$, in which **H** is the matrix of additive genetic relationships incorporating both pedigree and genomic information, $\sigma_{g}^{2}$ is the additive genetic variance and estimated from the pedigree-based BLUP (PBLUP), **e** is the vector of random residuals with distribution of N(0, $\text{I}\,\sigma_{e}^{2}$), in which **I** is the identity and $\sigma_{e}^{2}$ is the residual variance. **X**, **W**, and **Z** are the incidence matrixes associating **b, w,** and **g** with **y**, respectively.

The genotyped and ungenotyped animals were considered simultaneously based on a H matrix (Aguilar et al., 2010). The inverse of the H matrix was written as follows:

$${\text{H}}^{-1}=\left[\begin{array}{cc} 0 & 0\\ 0 & {\text{G}}_{w}^{-1}-{\text{A}}_{22}^{-1} \end{array}\right]+{\text{A}}^{-1}$$

where **A**^−**1**^ is the inverse of the numerator relationship matrix, ${\text{A}}_{22}^{-1}$ is the inverse of the pedigree-based relationship matrix for the genotyped animals, and ${\text{G}}_{w}^{-1}$ is the inverse of the genomic relationship matrix;, G weight markers were obtained by reciprocals of expected marker variance (VanRaden, 2008).

The SNP effects could be estimated by ssGWAS. The proportion of genetic variance explained by single SNP was calculated as follows:

$$\frac{\text{Var}\,\left({\text{Z}}_{j}\,{\hat{u}}_{j}\right)}{\sigma_{a}^{2}}\times 100\%$$

where $\sigma_{a}^{2}$ is the total genetic variance, **Z_j_** is a vector of the gene content of the jth SNP for all animals, and ${\hat{u}}_{j}$ is the estimated marker effect of the jth SNP.

##### Two-trait ssGWAS

According to the genetic correlation estimations, four body size traits with high genetic correlations (CC, AC, RW, and CW) were carried out using two-trait ssGWAS model.

$$\begin{array}{l} \left[\begin{array}{c} {\text{y}}_{1}\\ {\text{y}}_{2} \end{array}\right]\;=\left[\begin{array}{cc} X_{1} & 0\\ 0 & X_{2} \end{array}\right]\left[\begin{array}{c} b_{1}\\ b_{2} \end{array}\right]\;+\;\left[\begin{array}{c} \gamma_{1}\\ \gamma_{2} \end{array}\right]\left[\begin{array}{c} {\text{W}}_{1}\\ {\text{W}}_{2} \end{array}\right]\;+\;\left[\begin{array}{cc} Z_{1} & 0\\ 0 & Z_{2} \end{array}\right]\left[\begin{array}{c} g_{1}\\ g_{2} \end{array}\right]\\ \;+\left[\begin{array}{c} e_{1}\\ e_{2} \end{array}\right] \end{array}$$

where $\left[\begin{array}{c} {\text{y}}_{1}\\ {\text{y}}_{2} \end{array}\right]$ is the vector of observation values of trait I and II, *b*_*1*_ and **b_2_** are the vector of fixed effects of herd-year-season-sex of trait I and II, **X_1_** and **X_2_** are the incidence matrix associating **b_1_** and **b_2_** with **y_1_** and **y_2_**, $\left[\begin{array}{c} {\text{W}}_{1}\\ {\text{W}}_{2} \end{array}\right]$ is the vector of covariate of body weight of trait I and II, γ**_1_** and γ**_2_** are the regression coefficient associating **W_1_** and **W_2_**, $\left[\begin{array}{c} {\text{g}}_{1}\\ {\text{g}}_{2} \end{array}\right]$ is the vector of additive genetic effects of the two traits, following a normal distribution of *N*(**0**,**H**⊗**M**), where ***M*** = $\left[\begin{array}{cc} \sigma_{\mathrm{g1}}^{2} & \sigma_{\mathrm{g12}}^{2}\\ \sigma_{\mathrm{g12}}^{2} & \sigma_{\mathrm{g2}}^{2} \end{array}\right]$ is the additive genetic variance and covariance matrix of the two traits, **Z_1_** and **Z_2_** are the incidence matrix associating **g_1_** and **g_2_** with **y_1_** and **y_2_**, and $\left[\begin{array}{c} {\text{e}}_{1}\\ {\text{e}}_{2} \end{array}\right]$ is the vector of random errors with distribution of **N**(**0**,**I**⊗**R**), where **I** is the identity matrix and *R* = $\left[\begin{array}{cc} \sigma_{\mathrm{e1}}^{2} & \sigma_{\mathrm{e12}}^{2}\\ \sigma_{\mathrm{e12}}^{2} & \sigma_{\mathrm{e2}}^{2} \end{array}\right]$ is the residual variance and covariance matrix of the two traits.

In this study, for both the single-trait model and two-trait model of ssGWAS, blupf90 (Aguilar et al., 2011)was implemented to estimate genomic breeding values (GEBV), and afterwards, based on GEBV, SNP effects and *P*-values were estimated via postGSf90. The significance test of SNP effects was performed using two-sided *t*-test and the *P-*value of each marker was calculated as follows (Aguilar et al., 2019):

$${\text{P}}_{i}={\text{P}}_{t}\,\left(\frac{{\hat{u}}_{i}}{\sqrt{{\hat{\sigma }}_{i}^{2}\,/\,n}},\text{n}-1\right),$$

where **P_i_** is the distribution function of t distribution, ${\hat{u}}_{i}$ is ith SNP effect, ${\hat{\sigma }}_{i}^{2}$ is the genetic variance of ith SNP, and n is the number of animals with ith SNP. In addition, the proportion of genetic variance explained by the ith SNP could also be calculated as ${\hat{\sigma }}_{i}^{2}\,/\,\sigma_{g}^{2}$. Manhattan plots of SNP variance were obtained by the “qqman” R package(D. Turner, 2018).

In order to control false positives, the False Discovery Rate (FDR) (Benjamini and Hochberg, 1995; Weller et al., 1998) method for multiple testing was used as follows:

$$\mathrm{FDR}=m^{*}\,P_{\mathrm{Max}}/n$$

where m is the number of times to be tested and n is the number of significant SNPs at assigned FDR level, e.g., 0.05. *P*_*Max*_ is the genome-wide significance level empirical *P*-value of FDR adjusted. Based on the *P*-values of SNPs obtained by ssGWAS, the empirical *P*-value of FDR adjusted at the genome-wide significance level of 0.05 was calculated on each trait in this study.

### Identification of Candidate Genes

After identifying significant SNPs by ssGWAS, the genes located in the 50Kb downstream and 50 Kb upstream region of the significant SNPs were determined using BedTools (Quinlan and Hall, 2010) and pig reference gene annotation (^2^ Sus scrofa 11.1 genome version). The R package bioconductor^3^ was used to identify the related pathways and perform functional annotation. QTLdb^4^ was used to annotate significant SNPs located in previously mapped QTLs in pigs. R package ‘Cluster Profiler’ (Yu et al., 2012) was used to carry out Gene Ontology (GO) and Kyoto research on annotated candidate genes from the Encyclopedia of Genes and Genomes(KEGG) enrichment analysis.

## Results

### Genetic Correlations of Body Size Traits

Table 2 shows the genetic correlations of seven body size traits. The genetic correlations ranged from −0.286 to 0.840 with standard errors ranging from 0.028 to 0.106. Among the seven body size traits, chest circumference (CC) and abdominal circumference (AC), and chest width (CW) and rump width (RW) had the higher genetic correlations of 0.747 and 0.840 with standard errors of 0.055 and 0.028, respectively. The genetic correlations between other traits were lower than 0.3, and some traits were almost not genetically correlated with other traits, e.g., body length (BL) had a very low genetic correlation of −0.010, 0.03, −0.01, and 0.01 with body height (BH), CC, AC, and CW, respectively.

**TABLE 2:** Genetic correlations between seven body size traits.

Trait^1^	BL	BH	CC	AC	CW	RW	CBC
BL		−0.010(0.088)	0.033(0.092)	−0.014(0.092)	0.014(0.078)	−0.286(0.078)	0.206(0.078)
BH			0.171(0.104)	0.071(0.106)	−0.221(0.091)	−0.217(0.090)	−0.105(0.096)
CC				**0.747(0.055)**	0.255(0.093)	0.127(0.095)	0.197(0.096)
AC					0.153(0.096)	0.204(0.095)	0.202(0.096)
CW						**0.840(0.028)**	0.015(0.086)
RW							−0.032(0.085)
CBC							

*^1^BL, body length; BH, body height; CC, chest circumference; AC, abdominal circumference; CBC, cannon bone circumference; RW, rump width; CW, chest width. The two largest of all genetic associations are in bold. SE of estimates are in parentheses.*

### Identification of Significant SNPs Associated With Body Size Traits

**FIGURE 1:**
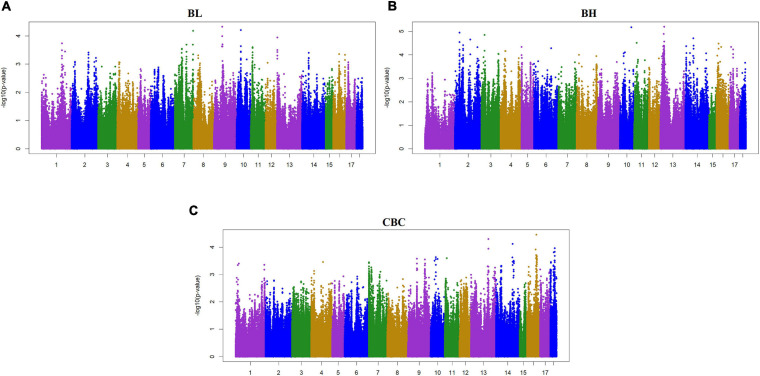
Manhattan plot of the genome-wide association study on three body size traits by using single-trait model ssGWAS. BL, Body length; BH, Body height; CBC, Cannon bone circumference. In the Manhattan plots, negative log10 *P*-values of the quantified SNPs were plotted against their genomic positions. The *x*-axis represents the chromosomes and the y-axis represents the observed −log10(*P*-value). Different colors indicate various chromosomes. Each trait has a significant threshold of FDR adjusted, for **(A)** BL, it was 9.26 × 10^–6^. Similarly, **(B)** BH was 1.08 × 10^–5^, and **(C)** CBC was 1.02 × 10^–5^.

**FIGURE 2:**
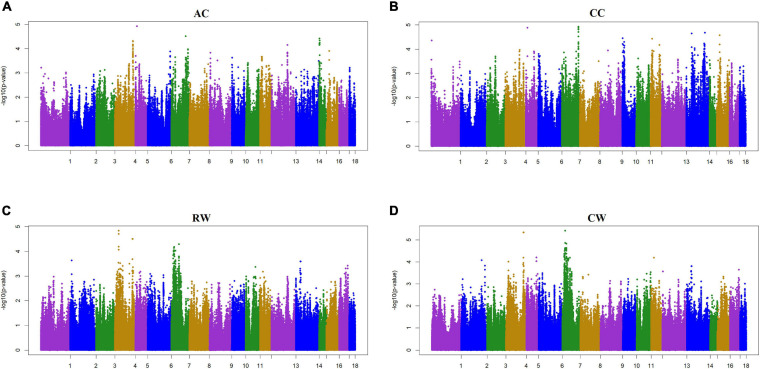
Manhattan plot of the genome-wide association study on four body size traits by using two-trait model ssGWAS. AC, Abdominal circumference; CC, Chest circumference; RW, Rump width; CW, Chest width. AC and CC are a pair of traits, RW and CW are a pair of traits. In the Manhattan plots, negative log10 *P*-values of the quantified SNPs were plotted against their genomic positions. The *x*-axis represents the chromosomes and the y-axis represents the observed –log10(*P*-value). Different colors indicate various chromosomes. Each trait has a significant threshold of FDR adjusted, for **(A)** AC, it was 9.74 × 10^–6^. Similarly, **(B)** CC was 1.05 × 10^–5^, **(C)** RW was 9.60 × 10^–6^, and **(D)** CW was 1.01 × 10^–5^.

Two criteria of *P*-value and SNP effect were respectively used to determine the SNPs associated with body size traits. As for the *P*-value, after the 0.05 significance level of the whole genome was adjusted, the *P*_*Max*_-values of FDR-based multiple tests were 9.26E−06 for BL, 1.08E−05 for BH, 1.02E−05 for CBC, 9.74E-06 for AC, 1.05E−05 for CC, 9.60E−06 for RW, and 1.01E−05 for CW. As shown in Table 3, a total of 88 significant SNPs was identified for seven body size traits. The Manhattan plots of the three traits BL, BH, and CBC using the single trait model are shown in Figure 1. For BL, a total of nine significant SNPs reached the genome-wide significance level, accounting for 0.0085% of the genetic variance in total. These significant SNPS were located on SSC1, SSC6, SSC8, SSC13, SSC14, SSC16, and SSC17. The SNP at SSC17:33632497 explained the largest genetic variance (0.0029%). For BH, only six SNPs were genome-wide significant, accounting for a total of 0.0123% of genetic variance. They were located on SSC3, SSC5, SSC14, and SSC16. The interpretation of ssc16: 886074 has the largest genetic variance (0.0082%). For CBC, there were 15 significant SNPs at the genome-wide level, which explained 0.0267% of the genetic variance, and the most significant SNPs were closely located on SSC1. For the two pairs of genetically correlated traits using the two-trait model, the Manhattan plots of AC and CC, and RW and CW are shown in Figure 2. In total, eight, 17, nine, and 24 SNPs were identified associated with AC, CC, RW, and CW, respectively, and these SNPs explained 0.0109, 0.0242, 0.0099, and 0.0281% of genetic variances for the corresponding traits. For each trait, the genetic variance explained by a single significant SNP was very small, the largest of which for each trait were 0.0051% (SSC5:15137502), 0.0067% (SSC4:64552365), 0.0038% (SSC9:2330339), and 0.0065% (SSC7:115471416), respectively. Although the genetic correlations existed among seven body size traits, no common significant SNPs were found.

**TABLE 3:** Significant SNPs and associated genes for seven body size traits.

Trait^1^	Chromosome	Position (bp)	*P*-value	SNP effect (%)	Gene	Distance	Gene function
BL	6	5671575	2.35E−07	0.00186	**CDH13**	+13217	cadherin 13
	1	6435744	1.5E−06	0.00017	NA		NA
	1	6472959	1.5E−06	0.00080	PRKN	+38323	parkin RBR E3 ubiquitin protein ligase
	17	33632497	2.62E−06	0.00289	ENSSSCG00000028461	−47405	signal regulatory protein alpha
	13	25520933	4.45E−06	0.00004	ULK4	−8396	unc-51 like kinase 4
	16	1276330	4.57E−06	0.00031	NA		NA
	14	137476010	6.47E−06	0.00054	NA		NA
	8	28933773	7.46E−06	0.00144	NWD2	−23316	NACHT and WD repeat domain containing 2
	13	166328893	8.39E−06	0.00039	NA		NA
BH	16	886074	2.84E−06	0.00817	CTNND2	+28239	alpha-2-macroglobulin like 1
	8	7942460	3.01E−06	0.00083	NA		NA
	3	26586077	4.62E−06	0.00117	CLEC19A	−45911	C-type lectin domain containing 19A
	5	62690928	6.5E−06	0.00004	A2ML1	−42827	alpha-2-macroglobulin like 1
	4	128701315	7.54E−06	0.00152	NA		NA
	14	33580513	9.85E−06	0.00060	HSPB8	+45615	heat shock protein family B (small) member 8
CBC	4	117759672	2.16E−07	0.00279	**CDC14A**	−34935	cell division cycle 14A
	13	182971424	1.83E−06	0.00420	**TMPRSS15**	−29625	transmembrane serine protease 15
	17	12868538	1.85E−06	0.00635	PSD3	−43049	pleckstrin and Sec7 domain containing 3
	1	1201299	2.3E−06	0.00025	ENSSSCG00000041157	−47914	NA
	1	1205821	2.3E−06	0.00018	ENSSSCG00000050693	−42855	NA
	1	1220233	2.3E−06	0.00039	ENSSSCG00000045916	−18409	NA
	1	1367723	2.3E−06	0.00064	ENSSSCG00000043714	+5537	NA
	18	21663467	0.000003	0.00400	GRM8	−14659	glutamate metabotropic receptor 8
	14	9698552	3.19E−06	0.00026	ENSSSCG00000049499	9436	NA
	5	7020488	3.46E−06	0.00023	PMM1	−49963	phosphomannomutase 1
	12	50490164	4.08E−06	0.00233	SPNS3	−47230	sphingolipid transporter 3 (putative)
	3	12869355	4.1E−06	0.00259	ENSSSCG00000036217	+18272	NA
	4	10221008	5.38E−06	0.00076	ASAP1	−37722	ArfGAP with SH3 domain, ankyrin repeat and PH domain 1
	2	124456560	5.76E−06	0.00036	PRR16	+6055	proline rich 16
	1	13806583	7.02E−06	0.00142	ENSSSCG00000004081	−2527	NA
**Trait^1^**	**Chromosome**	**Position (bp)**	***P*-value**	**SNP effect (%)**	**Gene**	**Distance**	**Gene function**
AC	8	3249196	1.88E−06	0.00134	AFAP1	−46660	actin filament associated protein 1
	9	14578071	2.31E−06	0.00128	NA		NA
	14	13670622	2.71E−06	0.00048	PRSS55	−4581	serine protease 55
	5	15137502	2.96E−06	0.00513	RHEBL1	−39837	RHEB like 1
	4	5362087	4.48E−06	0.00139	ENSSSCG00000044937	+36176	NA
	7	26363076	5.4E−06	0.00093	NA		NA
	14	43227411	5.77E−06	0.00024	ENSSSCG00000033385	−49062	KIAA1671 ortholog
	16	522752	6.96E−06	0.00014	**CTNND2**	−3796	catenin delta 2
CC	3	63528527	1.32E−07	0.00015	ENSSSCG00000008250	−41861	catenin alpha 2
	6	19429624	3.27E−07	0.00022	Metazoa_SRP	−49801	Metazoan signal recognition particle RNA
	1	3149903	7.78E−07	0.00047	PDE10A	−28771	phosphodiesterase 10A A
	6	120477523	1.95E−06	0.00173	FHOD3	−40874	formin homology 2 domain containing 3
	10	56219300	2.01E−06	0.00203	ITGB1	−46293	integrin subunit beta 1
	17	18990746	2.63E−06	0.00004	ANKEF1	−32351	ankyrin repeat and EF-hand domain containing 1
	17	18997949	2.63E−06	0.00008	ANKEF1	−37701	ankyrin repeat and EF-hand domain containing 1
	2	122228151	3.03E−06	0.00105	ENSSSCG00000051343	−14167	NA
	2	122235537	3.03E−06	0.00074	ENSSSCG00000051343	−21553	NA
	16	33630686	3.58E−06	0.00018	NA		NA
	16	33638300	3.58E−06	0.00079	NA		NA
	16	5533970	4.64E−06	0.00394	ENSSSCG00000016791	+16579	NA
	12	5297390	5.41E−06	0.00092	RNF157	−48707	ring finger protein 157
	4	64552365	5.7E−06	0.00666	ENSSSCG00000042029	−24706	NA
	10	43341283	7.22E−06	0.00064	CUBN	−39687	cubilin
	8	21799389	8.84E−06	0.00030	ENSSSCG00000050984	−18261	NA
	10	60737384	9.42E−06	0.00449	ENSSSCG00000011121	−24200	CUGBP Elav-like family member 2
RW	8	137165913	5.64E−07	0.00049	NA		NA
	9	2330339	2.74E−06	0.00381	SYT9	−11533	synaptotagmin 9
	1	38033383	4.19E−06	0.00149	NKAIN2	−12912	sodium/potassium transporting ATPase interacting 2
	3	63682227	5.03E−06	0.00010	NA		NA
	11	32555905	6.78E−06	0.00015	DIAPH3	+46764	diaphanous related formin 3
	16	48600234	7.1E−06	0.00118	ENSSSCG00000046085	−23005	NA
	16	48696355	7.1E−06	0.00155	ENSSSCG00000039883	+49947	NA
	1	100210738	7.97E−06	0.00095	**MAPK4**	+8278	mitogen-activated protein kinase 4
	1	100335688	7.97E−06	0.00017	**MAPK4**	−49706	mitogen-activated protein kinase 4
CW	8	132277288	8.17E−07	0.00009	PTPN13	−27877	protein tyrosine phosphatase non-receptor type 13
					MAPK10	−27281	mitogen-activated protein kinase 10
	7	115471416	9.52E−07	0.00653	PPP4R4	−18233	protein phosphatase 4 regulatory subunit 4
	14	37118119	1.09E−06	0.00289	ENSSSCG00000051786	−2275	NA
	14	37165658	1.09E−06	0.00051	ENSSSCG00000051786	−49874	NA
	14	37230969	1.09E−06	0.00039	ENSSSCG00000051786	−9755	NA
	2	80016213	2.07E−06	0.00192	COL23A1	−46865	collagen type XXIII alpha 1 chain
	14	139878474	2.34E−06	0.00437	TCERG1L	−46513	transcription elongation regulator 1 like
	12	49725382	2.67E−06	0.00112	TRPV1	−33424	transient receptor potential cation channel subfamily V member 1
	16	35012960	3.46E−06	0.00029	DDX4	−46102	DEAD-box helicase 4
	7	115132809	4.65E−06	0.00069	ENSSSCG00000002464	−31787	proline rich membrane anchor 1
	16	73800572	4.82E−06	0.00153	U6	+41611	U6 spliceosomal RNA
	16	73812833	4.82E−06	0.00172	U6	+29350	U6 spliceosomal RNA
	16	73816240	4.82E−06	0.00175	U6	+25343	U6 spliceosomal RNA
	9	107845695	5.74E−06	0.00014	ENSSSCG00000032905	−7136	NA
	8	76456715	6.42E−06	0.00057	ENSSSCG00000042273	−45304	NA
	3	131731345	6.59E−06	0.00001	ENSSSCG00000049751	−23407	NA
	3	131738702	6.59E−06	0.00016	ENSSSCG00000049751	−30764	NA
	3	131744661	6.59E−06	0.00016	ENSSSCG00000049751	−36723	NA
	3	131756951	6.59E−06	0.00025	ENSSSCG00000049751	−43896	NA
	3	131758601	6.59E−06	0.00024	ENSSSCG00000049751	−45546	NA
	5	61521505	7.76E-06	0.00033	ENSSSCG00000033403	−14845	C-type lectin domain family 7 member A-like
	16	67601687	8.2E-06	0.00052	ENSSSCG00000049229	−42425	NA
	7	92897102	4.65E−06	0.00109	**HMGA1**	+25885	high mobility group AT-hook 1
	15	11796106	9.96E−06	0.00074	NA		NA

*^1^BL, body length; BH, body height; CC, chest circumference; AC, abdominal circumference; CBC, cannon bone circumference; RW, rump width; CW, chest width; gene effect, proportion of genetic variance explained. The bold values are potential candidate genes that are selected based on gene function.*

**FIGURE 3:**
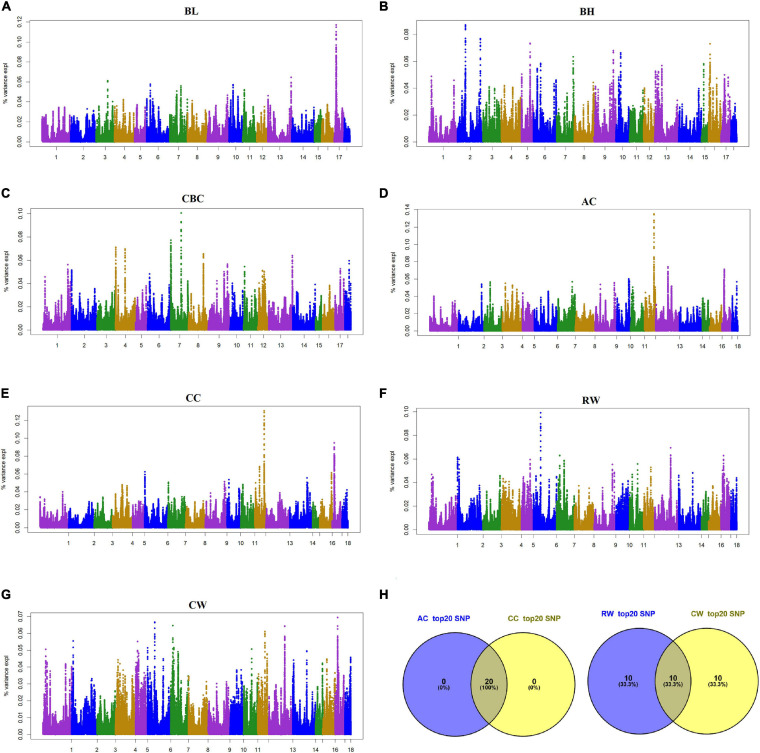
Manhattan plot of the genome-wide association study on seven body size traits and Venn plot of SNPs according to the contribution of SNP to genetic variance by using ssGWAS. BL, Body length; BH, Body height; CBC, Cannon bone circumference; AC, Abdominal circumference; CC, Chest circumference; RW, Rump width; CW, Chest width. BL, BH, and CBC were single-trait models, AC, CC, RW, and CW were two-trait models. AC and CC are a pair of traits, RW and CW are a pair of traits. In the Manhattan plots **(A–G)**, the proportion of genetic variance of the quantified SNPs were plotted against their genomic positions. The *x*-axis represents the chromosomes and the y-axis represents the percentage of SNP explaining the genetic variance. Different colors indicate different chromosomes. Venn plot **(H)** of SNPs for the two pairs of body size traits, AC and CC, RW, and CW are a pair of traits, respectively.

Considering the small contribution of the above significant SNPs to the genetic variance, the proportion of genetic variance explained by each SNP were also illustrated as shown in Figure 3 in this study. Top 20 SNPs with the largest genetic variance were selected for each trait (Table 4); SNPs for BL were located on SSC17, BH on SSC2, SSC5 and SSC16, and CBC on SSC7 and SSC4. SNPs with the largest genetic variance for AC and CC are located on SSC12, while those for RW and CW were on SSC6 SSC7, SSC13 and SSC17. For each body size trait, BL, BH, CBC, AC, CC, RW, and CW, the top 20 SNPs explained 2.01%, 1.56%, 1.63%, 2.39%, 2.32%, 1.54%, and 1.23% of the genetic variance, respectively. Interestingly, the top 20 SNPs for AC and CC were the same, while RW and CW shared half of the 20 SNPs. In total, 110 SNPs with a larger proportion of explanatory genetic variance were retained for further analysis (Supplementary Table 1).

**TABLE 4:** Overview of ssGWAS location for the percentage that explains the proportion of genetic variance.

Trait^1^	20 SNPs distributions of maximum effect	SNPs of the maximum effect	Top 20 SNPs effect (%)	Number of nearest gene	Candidate gene
BL	SSC17	17_7477978	0.117	4	
BH	SSC2, SSC5, SSC16	2_46827557	0.08.7	8	SIL1
CBC	SSC7, SSC4	7_55099416	0.101	17	TRAPPC9
AC	SSC12	12_53181656	0.128.	8	KDM6B CHD3
CC	SSC12	12_53169477	0.129	8	KDM6B CHD3
RW	SSC6, SSC7, SSC12, SSC13, SSC17	17_13172524	0.099	15	MUC13
CW	SSC6, SSC7, SSC13, SSC17	6_39554872	0.070	28	MUC13

*^1^BL, body length; BH, body height; CC, chest circumference; AC, abdominal circumference; CBC, cannon bone circumference; RW, rump width; CW, chest width. Gene effect, proportion of genetic variance explained.*

### Identification of Candidate Genes

All the significant SNPs identified by the two methods were annotated within the 50 Kb downstream and upstream region with reference to the Sus scrofa 11.1 genome assembly. According to the two methods of SNP significance and explained genetic variance, 88 and 110 SNPs were identified without overlapping, and 64 and 40 genes were found near these SNPs, with only two of them in common (Tables 1, 3). Six (*CDH13*, *CDC14A*, *TMRPSS15*, *CTNND2*, *MAPK4*, and *HMGA1*) and five (*SIL1*, *TRAPPC9*, *KDM6B*, *CHD3*, and *MUC13*) genes were found to be related to the corresponding body size traits by the two methods. The biological processes and pathways involved in these genes include calcium channel proteins, lipid metabolism, and cell proliferation.

## Discussion

### The Superiority of Imputation-Based WGS Data

Genotype marker density is one important factor affecting the efficiency of GWAS (Brondum et al., 2012). With the increase of marker density, the linkage disequilibrium between markers and the target trait QTL is increased, which is helpful for QTL detection. In previous studies, the advantages of whole genome sequencing data have been demonstrated (Wang L. et al., 2017). However, its high cost hampered the wide application of sequencing data. Genotype imputation proved efficient at imputing the SNP chip data to sequencing data with high accuracy (Hoze et al., 2013). Our results indicated that imputation-based WGS data dramatically improved the power of GWAS; among the significant SNPs identified in this study, only three out of the 88 significant SNPs were located in the PorcineSNP80 SNP chip, the remaining 85 loci were identified in the sequencing data. Moreover, among the 110 non-repeating loci screened by interpretation variance, 101 are new loci after imputation, which indicates that imputed WGS data adds a lot of useful information.

Increasing marker density could lead to high linkage disequilibrium (LD) to improve the resolution of gene mapping, although it may also be a burden (Joiret et al., 2019). Too high LD between markers will cause noise and increase false positive rates (Wang et al., 2010). One of the strategies to deal with such a dilemma is to pre-select SNP, which can be done via SNP selection, to only keep a set of SNPs that are mutually uncorrelated (Hazelett et al., 2016; Calus and Vandenplas, 2018). Therefore, we pruned SNPs according to the genome-wide sequence data to reduce the LD degree between SNPs, and retained the loci in the original 80K chip. In this study, 44003 out of the qualified 50179 SNPs in PorcineSNP80 chip according to the genotype quality control were retained, and the average linkage disequilibrium of the finally used 784, 267 SNPs is similar to that of the chip data. The average *r*^2^ was 0.191 and 0.195, respectively. This not only retains the original SNPs but also increases a large number of SNPS, and does not cause an increase of LD.

### The Advantage of ssGWAS

The single SNP regression model is widely used in GWAS to identify the association of SNP with traits of interest, although it usually yields a high false-positive rate due to ignoring the linkage disequilibrium between adjacent SNPs. Wang et al. (2012) proposed Single-step GWAS (ssGWAS) that combines all the data (genotype, phenotype, and pedigree information) in one step. It can simultaneously utilize all the markers compared with the single-marker regression genome-wide association analysis, resulting in higher power and accuracy (Wang et al., 2014). In addition, ssGWAS is able to use sliding windows to simultaneously analyze multiple SNPs to reduce errors (Braz et al., 2019; Guerra et al., 2019). Wang et al. (2012) reported that ssGWAS achieved an accuracy of 0.81 ± 0.02 using 1500 genotype animals, which was more accurate than single SNP regression model (Wang et al., 2012). Moreover, ssGWAS can utilize more individuals; the sample size in this study is not very large, but has a large amount of phenotypic data of ungenotyped animals. Compared with traditional GWAS, ssGWAS can make full use of this information, expand the sample size to a certain extent, improve the accuracy of SNP effect estimation, and further improve the efficiency of SNP identification.

In this study, different analysis models were used for body size traits. Two-trait GWAS model was used for traits with high genetic correlation, which can simultaneously use SNPs affecting two traits, resulting in reducing the false positives and improving the statistical power to detect genes (Chesler et al., 2005; Xu et al., 2009; Korte et al., 2012). However, for traits with low genetic correlation, the multi-trait model can easily lead to error information sharing across traits, reducing the lower accuracy of gene mapping as pointed out by Wang C. et al. (2017). We therefore used two traits with high genetic correlation in this study. In addition, we also conducted four-trait model for the GWAS on CC, AC, CW, and RW, in which the genetic correlation between CC and AC, and CW and RW was above 0.7, and the correlation between the other two traits were all lower than 0.3. The results showed that only 40% of significant SNPs (*P-*value) and 60% of large effects SNPs obtained by the four-trait model on average overlapped with those from the two-trait model for each trait. Moreover, the computation of the multi-trait model is demanding and difficult to converge.

#### The Determination of Significant SNP Using *P*-value or SNP Effect

Theoretically, the SNPs with the smallest *P*-values were supposed to explain relatively high proportions of genetic variance. Likewise, the SNPs with large effects should be significantly associated with the trait of interest. However, our results indicated that the SNPs with the smallest *P*-values did not have large effects, and there was no overlap between the top 20 SNPs with the smallest *P*-values and with the largest SNPs effects for each trait. Spearman correlation of *P*-values and effects of all SNPs showed that the rank correlations of seven body size traits were low (ranging 0.38–0.4), although they were significant. As pointed out by Aguilar et al. (2019), *P*-value explains whether markers have apparent effects that are seemingly different from 0 with statistical assessment, while SNP effect mainly explains part of the genetic variance, with no statistical assessment. The consistence of SNPs fitting well these two criteria could not be high due to the linkage disequilibrium of SNP, the impact of environment, and the interaction of SNPs. However, it provides an efficient way to determine the SNPs related to traits of interest. Therefore, in order to locate QTLs related to traits more accurately and comprehensively, this study identified significant SNPs from both *P-*value and SNP effect. The proportion of genetic variance explained by most of the significant SNPs was small (0.00004–0.00653%) for all traits, and the maximum genetic variance of all SNPs was also not large (0.0557–0.1205%), perhaps because too many SNPs were used in the sequencing data in this study, leading to a small effect of each related SNP for each trait. It also indicates that SNPs controlling body size traits are widely distributed on the genome, fitting the infinitesimal model well. It was reported that for complex traits such as height, action sites are widely distributed across the entire genome, indicating that almost all genes are involved in the regulation of height (Boyle et al., 2017).

Pleiotropic effects can lead to genetic correlation between traits. From the aspect of *P*-value, no overlap of significant SNPs associated with two genetic related trait pairs, AC and CC, and RW and CW, were detected in this study. However, more common SNPs with the largest effects (not statistically significant) were found in each pair of genetic related traits, e.g., the top 20 SNPs with largest effects for AC and CC were completely overlapped, and these SNPs were adjacent to each other and located near SSC12:53132997. Therefore, it is speculated that these SNPs constitute an important QTL and jointly affect AC and CC. Similarly, there may be QTLs associated with RW and CW around SSC6:39553559 and SSC13:135373704. In addition, we took 20 SNPs as a sliding window, and found that the top 20 windows with the largest genetic effects, respectively, for AC and CC were overlapped, as well as for RW and CW. The above results further reflect ‘one factor produces multiple effects’, suggesting that highly genetically related traits are probably regulated by the same QTL.

### Potential Candidate Genes for BL, BH, and CBC

The body length (BL) is an important index to investigate the breeding performance of animals. According to bioinformatics analysis, *CDH13* near SSC6:5671575 could be used as a candidate gene affecting body length. *CDH13* is a unique cadherin (Takeuchi et al., 2002) that regulates cell adhesion, signal transduction, and cell growth (Liu et al., 2016), and plays an important role in the formation of tissues and organs (Iotzova-Weiss et al., 2017). The ingestion and transfer of Ca will affect the bone development of the body for a long time (Kovacs, 2016), therefore, *CDH13* has a certain influence on the growth and development of the body. For BH, *SIL1* was found to be associated with this trait near SSC2:46827557. Proteomic studies showed that *SIL1* elevation alters the expression of proteins including crucial players in neurodegeneration, and abnormal expression of SIL1 has an impact on the morphology of the body, which can reduce the body size (Labisch et al., 2018). CBC reflects the physical quality of the animal, whether it is strong or not. There are three candidate genes associated with CBC: *CDC14A*, *TMPRSS15*, and *TRAPPC9*. *CDC14A* is widely expressed in eukaryotic cell biology of a special kind of highly conservative dual specificity phosphatase; a variety of studies from yeast to human somatic cells have shown that *CDC14* plays numerous roles, including in embryonic development and body size. *TMPRSS15* has an impact on the digestive efficiency of animals, and has been found to be associated with the formation of cholesterol in humans and with the development of fat and body weight in mice (Wang et al., 2016). *TMPRSS15* also has a higher variance ranking based on the SNP effect. The gene mutation of transporter particle complex 9 (*TRAPPC9*), a protein subunit of transporter particle II (*TRAPPII*), can lead to abnormal embryonic development and abnormal dietary behavior, and is associated with body mass index (Abbasi et al., 2017; Mbimba et al., 2018).

### Potential Candidate Genes for AC and CC

Abdominal circumference and CC are a pair of highly genetically related body size traits, which determine the body size of animals and are indicators of fatness and thinness. *CTNND2* was closely related to AC according to the *P*-value. Studies have shown that *CTNND2* participates in the regulation of cell proliferation and affects the body node number of zebrafish (Zhang et al., 2018). It is found that *KDM6B* and *CHD3* jointly affect AC and CC. KDM subfamily 6 enzymes B (*KAM6B*) plays an important role in the repression of developmental genes(Jones et al., 2018), and has a regulatory effect on chondrocyte differentiation, thus affecting bone growth and development (Dai et al., 2017). *CDH3* is a calcium-binding protein that is involved in calcium ion binding and protein binding and is associated with diseases such as malnutrition and developmental malformations. Studies have shown that *CHD3* regulates the developmental morphology of zebrafish heart, thereby affecting the abdominal circumference and body shape of zebrafish (Cho et al., 2018).

### Potential Candidate Genes for RW and CW

For RW and CW, *MUC13* was detected to affect both RW and CW. *MUC13* promotes cell proliferation and migration, inhibits apoptosis, reduces adhesion through a number of signaling pathways (Sheng et al., 2013), and has a certain effect on the absorption of intestinal nutrients, thus affecting the growth and development of bone and the organism. It was found that *MAPK4* and *HMGA1* affect RW and CW of pigs, respectively. *MAPK4* is mitogen-activated protein kinase 4, which is involved in the absorption and decomposition of sugars and the formation of fat, so it is related to obesity traits (Wu et al., 2016). *HMGA1* affects the expression of two *IGFBP* (insulin-like growth factor binding protein) protein species and plays an important role in cell growth and differentiation (Cleynen and Van de Ven, 2008; Hristov et al., 2010). Studies have shown that the deletion of *HMGA1* gene results in a significant decrease in the body size of mice (Federico et al., 2014). Moreover, a large number of studies have shown that *HMGA1* is related to the body size character of pigs. Ji et al. (2017) found *HMGA1* was a candidate gene affecting the body size of pigs through genome-wide association analysis. Zhang et al. (2014) found that *HMGA1* is expressed in pig limb cells and affects the growth and differentiation of chondrocytes. Because of the functional importance of *HMGA1* and several studies having shown that it is highly associated with body size traits, it is worth being verified in the future.

## Conclusion

In this study, among seven body size traits in pigs, CC and AC, and CW and RW were highly genetically correlated with correlations of 0.747 and 0.840, respectively. We implemented ssGWAS to identify SNPs associated with body size traits based on two aspects of *P*-value and the proportion of explanatory genetic variance of SNP. In total, 198 SNPs were identified associated with seven body size traits in Yorkshire pigs, correspondingly, 11 genes were related to body size traits, among which *HMGA1* could be worth being validated in further studies.

## Data Availability Statement

The data analyzed in this study is subject to the following licenses/restrictions: The ped and the map are not publicly available because the genotyped animals belong to commercial breeding companies, but are available from the corresponding author on reasonable request. Requests to access these datasets should be directed to XD, xding@cau.edu.cn.

## Ethics Statement

The animal study was reviewed and approved by the Institutional Animal Care and Use Committee (IACUC) of the China Agricultural University.

## Author Contributions

XD and CW conceived and supervised the study. HL, HS, and YL helped complete the imputation of the chip data and provide technical guidance. HL, YaJ, HS, and YiJ collected the samples and recorded the phenotypes. YaJ and YL extracted the DNA for genotyping. HL, FZ, YL, and YS contributed to the visualization of data. HL and XD wrote and revised the manuscript. All authors read and approved the manuscript.

## Conflict of Interest

The authors declare that the research was conducted in the absence of any commercial or financial relationships that could be construed as a potential conflict of interest.

## Footnotes

1 https://www.animalgenome.org/cgi-bin/QTLdb/SS/index

2 http://www.ensembl.org/Sus_scrofa/Info/Index/

3 http://www.bioconductor.org/

4 http://www.animalgenome.org/cgi-bin/QTLdb/SS/download?file=gbpSS_11.1
