# Supplementary material for: A Single-Step Genome Wide Association Study on Body Size Traits Using Imputation-Based Whole-Genome Sequence Data in Yorkshire Pigs
Source: Front Genet. 2021 Jul 2;12:629049. doi: 10.3389/fgene.2021.629049 (PMC8283822; doi:10.3389/fgene.2021.629049)
Supplement: Supplementary file 1 [file Table_1.DOCX]

**Supplementary Table 1.** **Each trait explains the 20 SNPs with the greatest genetic variance.**

| Trait^1^ | Chromosome | Position (bp) | SNP effect (%) | Gene | Gene function |
| --- | --- | --- | --- | --- | --- |
| BL | 17 | 7477978 | 0.1172 | ENSSSCG00000045345 | NA |
|  | 17 | 7467638 | 0.1170 | ENSSSCG00000045345 | NA |
|  | 17 | 7466396 | 0.1149 | ENSSSCG00000045345 | NA |
|  | 17 | 7466619 | 0.1104 | ENSSSCG00000045345 | NA |
|  | 17 | 7488442 | 0.1078 | ENSSSCG00000045345 | NA |
|  | 17 | 7478338 | 0.1071 | ENSSSCG00000045345 | NA |
|  | 17 | 7483444 | 0.1068 | ENSSSCG00000045345 | NA |
|  | 17 | 7390355 | 0.1034 | ZFP42 | ZFP42 zinc finger protein |
|  | 17 | 7461649 | 0.1024 | ENSSSCG00000045345 | NA |
|  | 17 | 7461584 | 0.1024 | ENSSSCG00000045345 | NA |
|  | 17 | 7304039 | 0.0971 | TRIML2 | tripartite motif family like 2 |
|  | 17 | 7305806 | 0.0948 | TRIML1 | tripartite motif family like 1 |
|  | 17 | 7392168 | 0.0944 | ZFP42 | ZFP42 zinc finger protein |
|  | 17 | 7436131 | 0.0937 | ZFP42 | ZFP42 zinc finger protein |
|  | 17 | 7459946 | 0.0927 | ENSSSCG00000045345 | NA |
|  | 17 | 7491963 | 0.0923 | ENSSSCG00000045345 | NA |
|  | 17 | 7436001 | 0.0907 | ZFP42 | ZFP42 zinc finger protein |
|  | 17 | 7395465 | 0.0901 | ZFP42 | ZFP42 zinc finger protein |
|  | 17 | 7388740 | 0.0900 | ZFP42 | ZFP42 zinc finger protein |
|  | 17 | 7411214 | 0.0868 | ZFP42 | ZFP42 zinc finger protein |
| BH | 2 | 46827557 | 0.0872 | PARVA | parvin alpha |
|  | 2 | 46842040 | 0.0865 | PARVA | parvin alpha |
|  | 2 | 46824879 | 0.0857 | PARVA | parvin alpha |
|  | 2 | 46818381 | 0.0857 | ENSSSCG00000044088 | NA |
|  | 2 | 46810711 | 0.0844 | ENSSSCG00000044088 | NA |
|  | 2 | 46800327 | 0.0837 | ENSSSCG00000044088 | NA |
|  | 2 | 46842660 | 0.0771 | PARVA | parvin alpha |
|  | 2 | 141143283 | 0.0769 | SIL1 | SIL1 nucleotide exchange factor |
|  | 2 | 141143283 | 0.0769 | SNORA74 | Small nucleolar RNA SNORA74 |
|  | 2 | 141170819 | 0.0761 | ENSSSCG00000047734 | NA |
|  | 2 | 46844298 | 0.0754 | PARVA | parvin alpha |
|  | 2 | 46849180 | 0.0751 | PARVA | parvin alpha |
|  | 2 | 46833492 | 0.0751 | PARVA | parvin alpha |
|  | 2 | 46836258 | 0.0749 | PARVA | parvin alpha |
|  | 2 | 46814696 | 0.0744 | ENSSSCG00000044088 | NA |
|  | 2 | 141156181 | 0.0741 | SIL1 | SIL1 nucleotide exchange factor |
|  | 2 | 46799382 | 0.0740 | ENSSSCG00000044088 | NA |
|  | 2 | 141172876 | 0.0740 | ENSSSCG00000047734 | NA |
|  | 5 | 83279164 | 0.0736 | ANO4 | anoctamin 4 |
|  | 5 | 83278416 | 0.0732 | ANO4 | anoctamin 4 |
|  | 16 | 5849688 | 0.0730 | RETREG1 | reticulophagy regulator 1 |
| CBC | 7 | 55099416 | 0.1008 | NA | NA |
|  | 7 | 55114854 | 0.0931 | NA | NA |
|  | 7 | 55074234 | 0.0926 | NA | NA |
|  | 7 | 55238376 | 0.0854 | NA | NA |
|  | 7 | 55147363 | 0.0849 | U6 | U6 spliceosomal RNA |
|  | 7 | 55066465 | 0.0849 | RHCG | Rh family C glycoprotein |
|  | 7 | 55181561 | 0.0812 | TICRR | TOPBP1 interacting checkpoint and replication regulator |
|  | 7 | 55256264 | 0.0773 | PEX11A | peroxisomal biogenesis factor 11 alpha |
|  | 7 | 55256264 | 0.0773 | WDR93 | WD repeat domain 93 |
|  | 7 | 55256264 | 0.0773 | KIF7 | kinesin family member 7 |
|  | 7 | 2207720 | 0.0773 | ENSSSCG00000050868 | NA |
|  | 7 | 55060037 | 0.0766 | ENSSSCG00000001841 | NA |
|  | 7 | 1339807 | 0.0752 | ENSSSCG00000043823 | NA |
|  | 7 | 1348275 | 0.0751 | ENSSSCG00000043823 | NA |
|  | 7 | 1346447 | 0.0749 | ENSSSCG00000043823 | NA |
|  | 7 | 1350355 | 0.0748 | ENSSSCG00000043823 | NA |
|  | 7 | 2205092 | 0.0726 | ENSSSCG00000050868 | NA |
|  | 7 | 1216905 | 0.0719 | GMDS | GDP-mannose 4,6-dehydratase |
|  | 7 | 1173595 | 0.0713 | GMDS | GDP-mannose 4,6-dehydratase |
|  | 4 | 3513374 | 0.0712 | TRAPPC9 | trafficking protein particle complex 9 |
|  | 4 | 3513374 | 0.0712 | KCNK9 | potassium two pore domain channel subfamily K member 9 |

| Trait^1^ | Chromosome | Position (bp) | SNP effect (%) | Gene | Gene function |
| --- | --- | --- | --- | --- | --- |
| AC | 12 | 53121986 | 0.0959 | CHD3 | chromodomain helicase DNA binding protein 3 |
|  | 12 | 53212072 | 0.0973 | ALOX15B | arachidonate 15-lipoxygenase B |
|  | 12 | 53212072 | 0.0973 | GUCY2D | guanylate cyclase 2D, retinal |
|  | 12 | 53213084 | 0.0973 | RNF227 | ring finger protein 227 |
|  | 12 | 53209927 | 0.1028 | CHD3 | chromodomain helicase DNA binding protein 3 |
|  | 12 | 53132997 | 0.1077 | KCNAB3 | potassium voltage-gated channel subfamily A regulatory beta subunit 3 |
|  | 12 | 53132997 | 0.1077 | TMEM88 | transmembrane protein 88 |
|  | 12 | 53202777 | 0.1108 | CHD3 | chromodomain helicase DNA binding protein 3 |
|  | 12 | 53179903 | 0.1130 | CHD3 | chromodomain helicase DNA binding protein 3 |
|  | 12 | 53199314 | 0.1189 | CHD3 | chromodomain helicase DNA binding protein 3 |
|  | 12 | 53144469 | 0.1190 | KDM6B | lysine demethylase 6B |
|  | 12 | 53181106 | 0.1191 | CHD3 | chromodomain helicase DNA binding protein 3 |
|  | 12 | 53144500 | 0.1195 | KDM6B | lysine demethylase 6B |
|  | 12 | 53141732 | 0.1196 | KDM6B | lysine demethylase 6B |
|  | 12 | 53137756 | 0.1197 | KDM6B | lysine demethylase 6B |
|  | 12 | 53150436 | 0.1204 | KDM6B | lysine demethylase 6B |
|  | 12 | 53146107 | 0.1257 | KDM6B | lysine demethylase 6B |
|  | 12 | 53188415 | 0.1278 | CHD3 | chromodomain helicase DNA binding protein 3 |
|  | 12 | 53169477 | 0.1279 |  | N-alpha-acetyltransferase 38, NatC auxiliary subunit |
| CC | 12 | 53122062 | 0.0922 | KCNAB3 | potassium voltage-gated channel subfamily A regulatory beta subunit 3 |
|  | 12 | 53122062 | 0.0922 | TMEM88 | transmembrane protein 88 |
|  | 12 | 53122062 | 0.0922 | CNTROB | centrobin, centriole duplication and spindle assembly protein |
|  | 12 | 53121986 | 0.0932 | ENSSSCG00000017963 | NA |
|  | 12 | 53131762 | 0.0934 | ENSSSCG00000017963 | NA |
|  | 12 | 53128937 | 0.0935 | ENSSSCG00000017963 | NA |
|  | 12 | 53212072 | 0.0992 | GUCY2D | guanylate cyclase 2D, retinal |
|  | 12 | 53212072 | 0.0992 | CHD3 | chromodomain helicase DNA binding protein 3 |
|  | 12 | 53213084 | 0.0992 | ALOX15B | arachidonate 15-lipoxygenase B |
|  | 12 | 53209927 | 0.1044 | KCNAB3 | potassium voltage-gated channel subfamily A regulatory beta subunit 3 |
|  | 12 | 53132997 | 0.1049 | CHD3 | chromodomain helicase DNA binding protein 3 |
|  | 12 | 53179903 | 0.1097 | CHD3 | chromodomain helicase DNA binding protein 3 |
|  | 12 | 53202777 | 0.1107 | CHD3 | chromodomain helicase DNA binding protein 3 |
|  | 12 | 53181106 | 0.1139 | KDM6B | lysine demethylase 6B |
|  | 12 | 53144469 | 0.1147 | KDM6B | lysine demethylase 6B |
|  | 12 | 53141732 | 0.1153 | KDM6B | lysine demethylase 6B |
|  | 12 | 53137756 | 0.1156 | KDM6B | lysine demethylase 6B |
|  | 12 | 53144500 | 0.1157 | KDM6B | lysine demethylase 6B |
|  | 12 | 53150436 | 0.1167 | CHD3 | chromodomain helicase DNA binding protein 3 |
|  | 12 | 53199314 | 0.1169 | KDM6B | lysine demethylase 6B |
|  | 12 | 53146107 | 0.1205 | KDM6B | lysine demethylase 6B |
| RW | 6 | 39554872 | 0.0992 | ENSSSCG00000050718 | NA |
|  | 6 | 39553559 | 0.0991 | ENSSSCG00000050718 | NA |
|  | 6 | 39559169 | 0.0955 | ENSSSCG00000050718 | NA |
|  | 6 | 39551923 | 0.0898 | ENSSSCG00000050718 | NA |
|  | 6 | 39556763 | 0.0874 | ENSSSCG00000050718 | NA |
|  | 6 | 39556962 | 0.0869 | ENSSSCG00000050718 | NA |
|  | 6 | 39559109 | 0.0859 | ENSSSCG00000050718 | NA |
|  | 6 | 39559635 | 0.0839 | ENSSSCG00000050718 | NA |
|  | 6 | 39551353 | 0.0800 | ENSSSCG00000050718 | NA |
|  | 6 | 39561973 | 0.0750 | ENSSSCG00000050718 | NA |
|  | 6 | 39543451 | 0.0702 | ENSSSCG00000050718 | NA |
|  | 13 | 135373704 | 0.0695 | MUC13 | mucin 13, cell surface associated |
|  | 13 | 135375265 | 0.0694 | ENSSSCG00000011859 | NA |
|  | 6 | 39562389 | 0.0670 | U6 | U6 spliceosomal RNA |
|  | 13 | 135363147 | 0.0632 | ENSSSCG00000011859 | NA |
|  | 7 | 15808427 | 0.0630 | ENSSSCG00000040178 | NA |
|  | 17 | 13172524 | 0.0629 | PSD3 | pleckstrin and Sec7 domain containing 3 |
|  | 7 | 15808323 | 0.0629 | E2F3 | E2F transcription factor 3 |
|  | 7 | 15812286 | 0.0629 | E2F3 | E2F transcription factor 3 |
|  | 7 | 15810514 | 0.0628 | E2F3 | E2F transcription factor 3 |
| CW | 17 | 13172524 | 0.0695 | PSD3 | pleckstrin and Sec7 domain containing 3 |
|  | 6 | 39554872 | 0.0667 | U6 | U6 spliceosomal RNA |
|  | 6 | 39553559 | 0.0664 | ENSSSCG00000050718 | NA |
|  | 7 | 12033680 | 0.0646 | NA | NA |
|  | 13 | 135373704 | 0.0644 | ENSSSCG00000011859 | NA |
|  | 17 | 13172648 | 0.0643 | PSD3 | pleckstrin and Sec7 domain containing 3 |
|  | 13 | 135375265 | 0.0641 | ENSSSCG00000011859 | NA |
|  | 6 | 39559169 | 0.0630 | ENSSSCG00000050718 | NA |
|  | 17 | 13169187 | 0.0627 | PSD3 | pleckstrin and Sec7 domain containing 3 |
|  | 12 | 40632539 | 0.0611 | NA | NA |
|  | 12 | 40649273 | 0.0600 | NA | NA |
|  | 6 | 39551923 | 0.0596 | ENSSSCG00000050718 | NA |
|  | 12 | 40631633 | 0.0594 | NA | NA |
|  | 12 | 40606648 | 0.0591 | NA | NA |
|  | 6 | 39556763 | 0.0584 | ENSSSCG00000050718 | NA |
|  | 17 | 13173183 | 0.0584 | ENSSSCG00000007034 | NA |
|  | 13 | 135363147 | 0.0582 | ENSSSCG00000011859 | NA |
|  | 12 | 40627770 | 0.0582 | NA | NA |
|  | 6 | 39556962 | 0.0577 | ENSSSCG00000050718 | NA |
|  | 13 | 135403430 | 0.0577 | MUC13 | mucin 13, cell surface associated |

Note: ^1^BL**=**body length; BH= body height, CC=chest circumference; AC=abdominal circumference; CBC= cannon bone circumference; RW= rump width; CW=chest width; SE of estimates are in parentheses. gene effect= proportion of genetic variance explained
